# A proteomics study of the response of North Ronaldsay sheep to copper challenge

**DOI:** 10.1186/1746-6148-2-36

**Published:** 2006-12-27

**Authors:** Deborah M Simpson, Ali Mobasheri, Susan Haywood, Robert J Beynon

**Affiliations:** 1 Department of Veterinary Preclinical Sciences, Faculty of Veterinary Science, University of Liverpool, Crown Street, Liverpool L69 7ZJ, UK; 2 Department of Veterinary Pathology, Faculty of Veterinary Science, University of Liverpool, Crown Street, Liverpool L69 7ZJ, UK; 3 School of Veterinary Medicine and Science, University of Nottingham, Sutton Bonnington Campus, Loughborough LE12 5RD, UK

## Abstract

**Background:**

The objective of this proteomics study was to identify proteins that changed expression as a result of copper challenge in the uniquely copper sensitive North Ronaldsay sheep and further, to compare those changes in expression with the more copper tolerant Cambridge breed. Such data gives us a proteome-centered perspective of the pathogenesis of copper-induced oxidative stress in this breed.

**Results:**

Many proteins respond to copper challenge, but this study focuses on those exhibiting a differential response between the two breeds, related to liver copper content. As copper accumulated in the tissue, the pattern of expression of several proteins was markedly different, in North Ronaldsay sheep as compared to the Cambridge breed.

**Conclusion:**

The pattern of changes was consistent with the greatly enhanced susceptibility of North Ronaldsay sheep to copper-induced oxidative stress, focused on mitochondrial disturbance with consequent activation of hepatic stellate cells. The expression profiles were sufficiently complex that the response could not simply be explained as a hypersensitivity to copper in North Ronaldsay sheep.

## Background

Copper is an essential trace element and the cofactor of several enzymes involved in a wide variety of physiological processes. However by virtue of its ability to participate in single-electron transfer reactions, copper can also generate reactive oxygen species which can be highly damaging to cell membranes and biomolecules. For this reason copper homeostasis has evolved as a tightly regulated process with an array of transporter and chaperone proteins regulating the uptake and excretion of copper, preventing its free accumulation within cells. Interruption or modification of this homeostasis can cause disease. A group of clinically and pathologically indistinguishable copper-associated diseases occur in infancy and childhood and are named Indian childhood cirrhosis (ICC), idiopathic copper toxicosis (ICT) and endemic Tyrolean infantile cirrhosis (ETIC). These life-threatening disorders, characterised by a florid pericellular fibrosis and cirrhosis have been linked with exogenous copper and also a genetic predisposition in the Tyrol [1] and in N. Germany [2]. ICC and ETIC have been attributed to excess copper intake from the use of brass or copper vessels to prepare infant feed [3] or well water with low pH [1]. The aetiology of sporadic cases of ICT in Germany, implicated the absence or short duration of breast feeding and the substitution of formula milk made with water at pH < 6.5 contaminated with copper 3–26 mg/L (WHO requirements <2 mg/L)[4].

The North Ronaldsay (NR), a primitive breed of sheep, has been identified as a possible model of these non-Wilsonian infant copper toxicoses [5]. Sheep generally are intolerant of dietary copper excess due to their impaired ability to excrete copper in the bile, and liver copper accumulation with ensuing toxicity is well recognised. The NR breed manifest this species propensity in extreme form and are the most copper-sensitive mammals known. This ancient and isolated breed of sheep occupy an ecological niche on the foreshore of North Ronaldsay island in the Orkney archipelago, where they have been exposed to a copper-impoverished diet, mainly seaweed [Cu < 5 ppm], for so long that they have evolved mechanisms to maintain adequate copper reserves [6]. In so doing they appear to have compromised their homeostatic mechanism, such that under copper-replete conditions they accumulate copper to excess with ensuing toxicity. NR sheep and their cross-breeds absorb more dietary copper than other breeds of domesticated sheep, proving that this trait is heritable [7]. In addition to an ecogenetic aetiology, Ronaldsay copper toxicosis (RCT) has a pathomorphology distinct from copper poisoning in domesticated sheep [8], but similar to the infant copper hepatic toxicoses [5]. Recent studies with artificially fed copper-supplemented North Ronaldsay lambs confirmed the unique status of this animal model with regard to the childhood disorders [9].

Genetic factors must ultimately underlie these differences in response to copper between different breeds of sheep, but at the metabolic level it is proteins that are responsible for its expression. An exploration of the effect of copper challenge on protein expression in the copper sensitive North Ronaldsay sheep compared with a more copper-tolerant breed, the Cambridge, would aid understanding of their respective pathophysiologies and contribute to understanding of copper toxicosis in humans. The study of Cambridge sheep has been completed and the pattern of biochemical changes was consistent with an early adaptive response to copper challenge, followed by an impaired ability to compensate for an increasing copper burden, initiating oxidative stress-induced injury in the prehaemolytic phase. These findings are reported in full elsewhere [10] and provide the basis from which to compare the response of North Ronaldsay sheep which are the main subject of this investigation. An evaluation of the pathological changes at the ultrastructural level in the two breeds of sheep has formed a parallel study [11].

## Results and discussion

**Figure 1:**
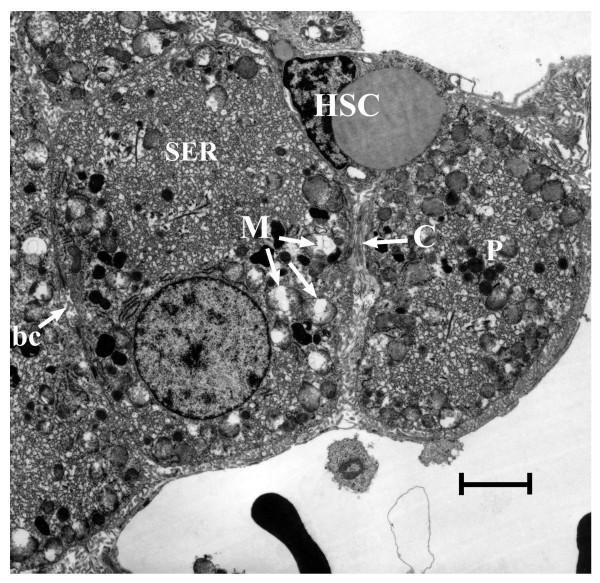
**Electron micrograph of NR sheep liver (Cu 800 μg/g)**. Hepatocytes (mid zone) show mitochondrial distention and frequent gross swelling with rupture (M); peroxizomes (P) and hypertrophied smooth endoplasmic reticulum (SER). Bile canaliculus (bc). Hepatic stellate cell (HSC) with collagen fibrils (C) penetrating intercellular space and separating adjacent hepatocytes. Bar = 2 μm.

Most studies of the cellular response to oxidative stress have been carried out *in vitro *with cells in culture. Here we describe a proteomics study of a unique intact animal model, the North Ronaldsay sheep that is acutely sensitive to copper-induced oxidative stress. The response to copper challenge in these animals has been compared to a copper tolerant breed, the Cambridge. Despite a 10-fold higher dosage of copper administered to the Cambridge (155 mg/Kg) compared with NR sheep (15 mg/Kg), the comparative liver pathology differed both qualitatively and quantitatively [11]. In particular, NR sheep show early severe mitochondrial damage with ballooning accompanied by modulation of hepatic stellate cell phenotype towards extracellular matrix (collagen) production, the precursor to fibrosis/cirrhosis (Fig 1).

### Liver copper and tissue microarrays

**Figure 2:**
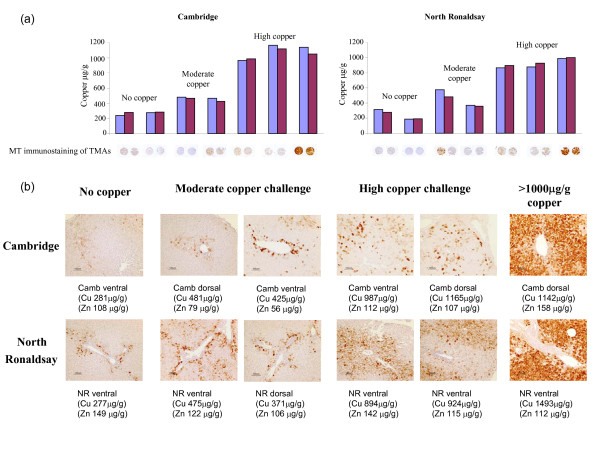
**Tissue microarray analysis of metallothionein and correlation with copper burden**. **(a) **Histogram representation of copper concentrations in the dorsal (blue bars) and ventral (pink bars) liver lobes of individual animals used in the study. The corresponding 2 mm tissue cores punched from formalin-fixed paraffin-embedded sections of the same liver tissue are shown below each bar. **(b) **Zoomed-in regions of individual tissue cores and the copper and zinc concentrations of the corresponding liver lobes are shown underneath. Briefly, tissue microarray (TMA) slides were dewaxed, re-hydrated and processed according to the protocol described in the Envision dual labelling kit. TMAs were incubated with a 1:250 dilution of anti-MT antibody, in blocking solution (1%(w/v) BSA in TBS pH7.6) overnight at 4°C. TMAs were incubated with a peroxidase-labelled polymer conjugated to goat anti-mouse and goat anti-rabbit immunoglobulins then developed using a DAB buffered substrate. TMA slides were counterstained with haematoxylin, dehydrated and photographed (magnification ×10).

Tissue copper and zinc concentrations were determined by inductively-coupled plasma mass spectrometry (ICP-MS) of both dorsal and ventral liver lobes for individual animals used in this study. In all animals the concentrations of each metal in both liver lobes was similar. The expression of metallothionein (MT) was assessed by immunohistochemical analysis of tissue microarrays (TMAs). Figure 2a shows a histogram representation of the copper concentration of both dorsal and ventral liver lobes for individual animals used in this study. The corresponding 2 mm tissue cores from a TMA probed with anti-MT antibody is aligned below each bar. A zoomed area from each of the cores (x10 magnification) is shown in Fig 2b. Metallothionein reactivity was expressed within isolated hepatocytes in the periportal lobular zones of the controls (low copper) of both sheep breeds. With increased copper loading, MT immunoreactivity increased periportally until at high copper challenge with one exception (Cambridge) all tissues above a threshold of 1000 μg/g copper exhibited strong panlobular staining for metallothionein. Zinc concentrations did not increase with increased MT immunoreactivity confirming that MT expression was consistent with liver copper. The TMA results indicate that MT immunoreactivity was expressed most consistently and strongly in NR sheep and although this may reflect the lower copper threshold of MT induction may also reflect the high potential for copper-induced oxidative stress in this breed.

### Differential protein expression

**Figure 3:**
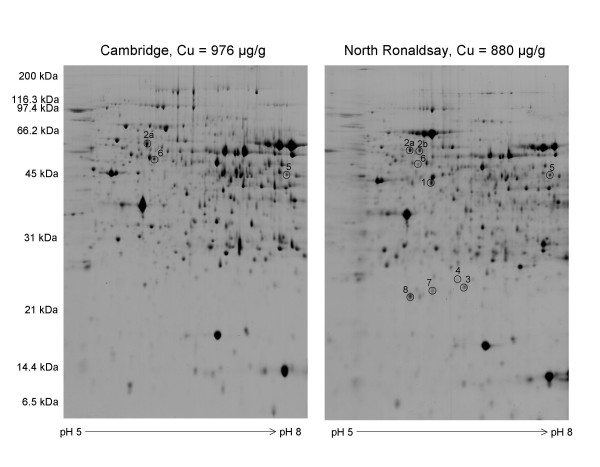
**Two-dimensional gel electrophoresis of soluble liver proteins isolated from Cambridge and North Ronaldsay sheep following copper challenge**. Proteins were separated in the first dimension on pH 5–8 immobilized pH gradient strips, followed by SDS-PAGE on 12.5% (w/v) gels. Gels were stained with Coomassie Brilliant Blue G-250. Proteins whose expression was statistically significant between both sheep breeds as determined by Students t-test are numbered and the identities of these proteins are given in Tables 1 and 2. The copper level in each individual liver (averaged across dorsal and ventral lobes, μg/g dry weight) is indicated on the top of each panel.

A study of differential protein expression using comparative 2-D gel analysis and a combination of MALDI-ToF and tandem MS analysis was carried out on the same tissues in order to evaluate the impact of copper-induced oxidative stress on protein expression in the two breeds of sheep. Proteins were resolved in the first dimension by isoelectric focusing across the pH range 5–8 which gives excellent resolution of large numbers of liver proteins. In the second dimension proteins were electrophoresed in a linear 12.5% (w/v) polyacrylamide gel which resolved proteins over a mass range of 200 kDa -6.5 kDa. Representative 2-D gels of soluble liver proteins isolated from copper-challenged North Ronaldsay and Cambridge sheep indicate both the complexity and subtle differences in the protein patterns over this pI range [see Additional file 1]. The comparative 2-D gel analysis revealed many differences in the 2-D protein maps of soluble liver proteins between Cambridge and North Ronaldsay sheep (Figure 3). The majority of the differences were breed rather than copper-responsive changes in protein expression. The copper responsive changes in North Ronaldsay and Cambridge sheep are summarised in Tables 1 and 2 respectively and their location on the 2-D gel is indicated in Figure 3. A list of expression differences related to breed are also supplied [see Additional file 2]. It should however be borne in mind that whilst this study is a global proteomics study of soluble liver proteins only those proteins identified by the software as being differential expressed were targeted for excision and subsequent identification by MS. It is possible that a protein may be present in one or more locations on the 2-D gel and that in only one of these locations (a possible modified form) differential expression is observed between the two breeds of sheep. In Additional file 2 there are two instances where duplicate entries for a protein have been entered (60 kDa heat shock protein and acyl CoA dehydrogenase). Both entries are from individually quantitated spots.

**Table 1:** Identification of copper-responsive proteins in North Ronaldsay sheep. 2-D gels for each animal were run in triplicate and placed in analysis sets. Statistical analysis sets were created from two copper-challenged Cambridge (n = 6 gels) and North Ronaldsay (n = 6 gels) animals and statistically significant changes in protein expression were identified by Student's t-test at p ≤ 0.05.

Protein Identification	Accession	Species	Mwt (kDa)	Mowse score	Blast score	E value	Seq coverage	Fold change
1. Cathepsin D	[Swiss-Prot:Q9MZS8]	Sheep	39.8		33	0.78	VSSLPQVTLK	4
2b Epoxide hydrolase	[PIR:A47504]	Mouse	62.6		35	0.1	(AV)ASLNTPFM	N/A
	[EMBL:CAA46211.1]	Rat			41	0.002	YQLPALAQAGFR	
3. Ferritin light chain	[Uniref:O46415]	Cow	19.9	112			31%	4
7. Peroxiredoxin 3 (SP-22)	[PIR:JC2258]	Cow	21.7	74			30%	10.0
		Rat	28.3		38	0.031	HLSVNDLPVGR	
8. Plasma retinol binding protein	[PIR:S13186]	Cow	21.4	130			30%	2.8

2-D gels for each animal were run in triplicate and placed in analysis sets. Statistical analysis sets were created from two copper-challenged Cambridge and North Ronaldsay animals and statistically significant changes in protein expression were identified by Student's t-test at p ≤ 0.05.

**Table 2:** Identification of copper-responsive proteins in Cambridge sheep. 2-D gels for each animal were run in triplicate and placed in analysis sets. Statistical analysis sets were created from two copper-challenged Cambridge (n = 6 gels) and North Ronaldsay (n = 6 gels) animals and statistically significant changes in protein expression were identified by Student's t-test at p ≤ 0.05.

Protein Identification	Accession	Species	Mwt (kDa)	Mowse score	Blast score	E value	Seq coverage	Fold change (%)
2a. Epoxide hydrolase	[PIR:A47504]	Mouse	62.6	-	29	9.3	(LG)ASLNTPFMQAVP	3
	[EMBL:AAS68016]	Pig		-	25	238	SSDETFLTVSR	
		Several			25	142	(YQ)LPALAQ(FQ)R	
4. Heat shock protein Hsp 27	[Swiss-Prot:JC4244]	Dog	22.9	86			29%	N/A
5. Cytosolic NADP^+^-dependent isocitrate dehydrogenase	[trEMBL:Q6XUZ5]	Sheep	47.2	272			61%	N/A
6. Methionine adenosyl transferase	[Swiss-Prot:S06114]	Rat	44.2	129			27%	2.6

2-D gels for each animal were run in triplicate and placed in analysis sets. Statistical analysis sets were created from two copper-challenged Cambridge and North Ronaldsay animals and statistically significant changes in protein expression were identified by Student's t-test at p ≤ 0.05.

**Figure 4:**
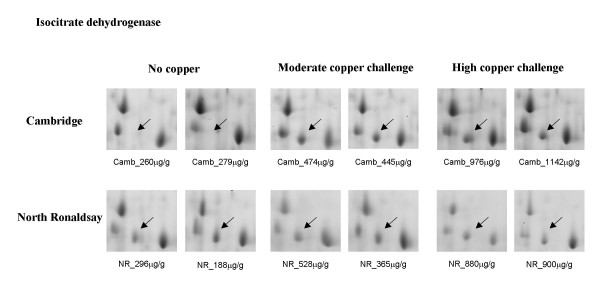
**Differential expression of NADP^+^-dependent isocitrate dehydrogenase in Cambridge and North Ronaldsay sheep at both moderate and high copper challenge**. The region of the 2-D gel encompassing a protein identified as cytosolic NADP^+^-dependent isocitrate dehydrogenase by MALDI-ToF MS and peptide mass fingerprinting is shown for two control animals, and two copper-challenged animals at both moderate and high copper loading. The copper level in each individual liver (mean value of dorsal and ventral lobes), determined by ICP-MS is indicated.

**Cytosolic NADP^+^-dependent isocitrate dehydrogenase **(IDH) was induced in the Cambridge sheep following both moderate and high copper challenge but was detected in both control and copper challenged North Ronaldsay (NR) sheep (Figure 4). Both mitochondrial and cytosolic IDH are induced in response to oxidative stress [12,13]. Moreover, IDH is essential for efficient glutathione recycling, and the maintenance of the cellular redox state [12,13]. The presence of IDH in the cytosol of unchallenged NR sheep liver would suggest that the liver tissue had previously been 'primed' by factors which would induce expression of IDH which in turn suggests an increased susceptibility to oxidative stress in NR sheep.

**Figure 5:**
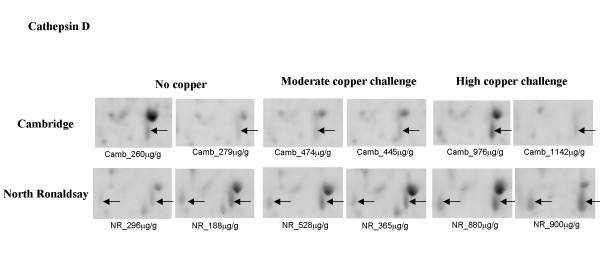
**Differential expression of cathepsin D in Cambridge and North Ronaldsay sheep at both moderate and high copper challenge**. The region of the 2-D gel encompassing a protein identified as cathepsin D by MALDI-ToF MS and peptide mass fingerprinting is shown for two control animals, and two copper-challenged animals at both moderate and high copper loading. The copper level in each individual liver (mean value of dorsal and ventral lobes), determined by ICP-MS is indicated.

**Cathepsin D **was increased in copper-challenged NR sheep but not in the Cambridge breed (Figure 5). The protein was identified in two locations in NR gels by MALDI-ToF MS, and spot intensity in both these locations was increased following copper challenge. Cathepsin D is a major cellular aspartic protease that has numerous functions within the lysosomal compartment. Lysosomal rupture has been implicated in necrosis, the predominant mode of cell death in the copper-challenged NR sheep livers [11]. In the oxidative environment induced by copper challenge it is probable that membrane damage to the lysosome occurs resulting in a release of cathepsin D into the cytosol which in turn activates caspase-3 leading to mitochondrial rupture. This is consistent with the model proposed whereby free copper ions in the lysosomes undergo redox cycling with cysteine to form reactive oxygen species from hydrogen peroxide formed by autophagocytosed damaged mitochondria or from Cu^1+ ^autooxidation. Lipid peroxidation catalysed by Cu^2+ ^or reactive oxygen species could thus contribute to lysosomal 'leakiness'resulting in the intracellular release of lysosomal proteases and phospholipases [14]. An ultrastructural study of copper-challenged NR liver revealed the prominence of Kupffer cells, the latter phagocytic cell comprise about 2% of the liver cell population and yet are estimated to contain approximately 26% of the liver's lysosomes which may in part account for the increased levels of cathepsin D observed. In addition cathepsin D is upregulated in activated hepatic stellate cells (HSCs) both *in vitro and in vivo *[15] and activated HSC's were a feature of the liver pathology of NR sheep. Transdifferentiation of HSCs into myoblast-like cells with the production of extracellular matrix (collagen) was observed exclusively in NR sheep [11].

**Figure 6:**
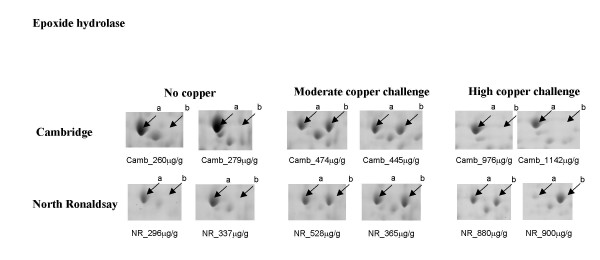
**Differential expression of epoxide hydrolase in Cambridge and North Ronaldsay sheep at both moderate and high copper challenge**. The region of the 2-D gel encompassing a protein identified as epoxide hydrolase by sequence tags generated by tandem MS is shown for two control animals, and two copper-challenged animals at both moderate and high copper loading. The copper level in each individual liver (mean value of dorsal and ventral lobes), determined by ICP-MS is indicated.

**Epoxide hydrolase**, which plays a role in the removal of xenobiotics [16,17], was identified at two locations on the 2-D gel (Figure 6). The change in expression of this protein occurred following copper challenge. A number of possibilities exist for the presence of the epoxide hydrolase isoform, one of these being that the protein is induced following copper challenge, alternatively copper challenge may mediate a post-translational modification of the protein. Interestingly, in the Cambridge sheep at high copper challenge, epoxide hydrolase expression is similar to that of the control gels with the absence of the second isoform (Figure 6, spot b). However, in the NR sheep expression of the isoform is maintained moreover, in one of the sheep only the second isoform is present which would support the hypothesis of post translational modification of the protein. In addition an inverse relationship exists between the spot quantities of both epoxide hydrolase isoforms which is further evidence of post-translational modification. The presence of a single gene in the human genome coding for this protein is also supportive of post-translational modification. The absence of the modified protein at high copper challenge in the Cambridge sheep lends support to a breed difference in the ability of the liver to restore homeostasis, the Cambridge breed having an increased capacity to do so.

**Figure 7:**
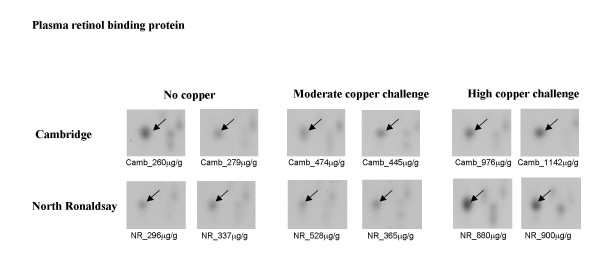
**Differential expression of plasma retinol binding protein in Cambridge and North Ronaldsay sheep at both moderate and high copper challenge**. The region of the 2-D gel encompassing a protein identified as plasma retinol binding protein by MALDI-ToF MS and peptide mass fingerprinting is shown for two control animals, and two copper-challenged animals at both moderate and high copper loading. The copper level in each individual liver (mean value of dorsal and ventral lobes), determined by ICP-MS is indicated.

**Plasma retinol binding protein **(RBP), responsible for the circulation of retinol in the plasma was increased between 1.5 and 2-fold in Cambridge sheep following high copper challenge. By comparison in NR sheep plasma RBP was increased substantially to between 2 and 3-fold compared to unchallenged controls (Figure 7). Activation of HSCs, a feature of NR sheep would deplete retinol in these cells [9,11]. The synthesis of RBP regulates retinol release from the liver; mobilisation of retinol from HSCs would therefore require a concomitant increase in RBP.

**Figure 8:**
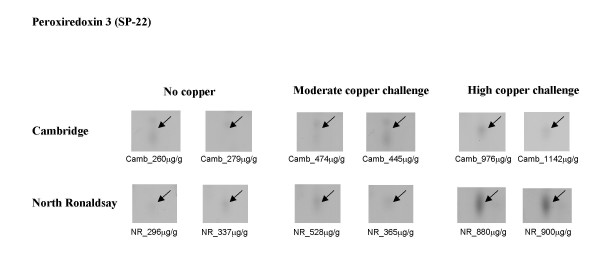
**Differential expression of peroxiredoxin III (SP-22) in North Ronaldsay sheep at both moderate and high copper challenge**. The region of the 2-D gel encompassing a protein identified as peroxiredoxin III (SP-22) by MALDI-ToF MS and peptide mass fingerprinting and by sequence tags generated by tandem MS is shown for two control animals, and two copper-challenged animals at both moderate and high copper loading. The copper level in each individual liver (mean value of dorsal and ventral lobes), determined by ICP-MS is indicated.

**Peroxiredoxin III (SP-22) **a member of the peroxiredoxin family of enzymes is a small (22 kDa) protein localised in the mitochondrial matrix. SP-22 plays a role in the protection of several free radical-sensitive enzymes from inactivation by metal-catalysed, free-radical-generating systems [18,19]. Expression of SP-22 was dramatically increased in the cytosol of NR sheep at high compared with moderate copper challenge (Figure 8). This observation was coincident with ballooning and rupture of mitochondria observed only in NR sheep [11].

**Figure 9:**
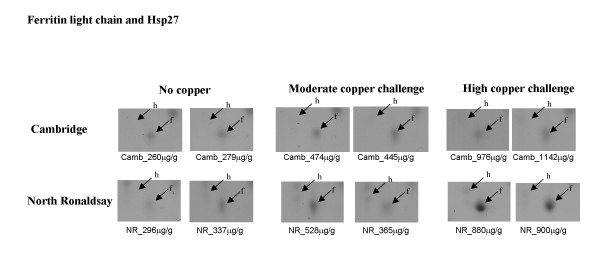
**Differential expression of ferritin light chain and Hsp27 in North Ronaldsay sheep at both moderate and high copper challenge**. The region of the 2-D gel encompassing a protein identified as ferritin light chain (f) and Hsp27 (h) by MALDI-ToF MS and peptide mass fingerprinting is shown for two control animals, and two copper-challenged animals at both moderate and high copper loading. The copper level in each individual liver (mean value of dorsal and ventral lobes), determined by ICP-MS is indicated.

**Ferritin light chain**, an iron storage protein, was up-regulated exclusively in the NR sheep (Figure 9). Accumulation of iron deposits is a pathological feature of chronic liver diseases such as cirrhosis and hepatocellular carcinoma as well as accompanying metabolic disturbance. Caeruloplasmin (a multicopper oxidase) is an essential ferroxidase critical for iron export. Disturbance of copper homeostasis and ensuing oxidative stress may impede the efficient efflux of iron by caeruloplasmin.

**Methionine adenosyl transferase**, identified by MALDI-ToF MS and peptide mass fingerprinting was more than 2-fold higher in abundance (p < 0.05) in the gels prepared from the Cambridge versus NR liver tissue however, spot quantitation in control versus copper challenged Cambridge sheep showed no copper-responsive increase in the expression of this protein, indicative of a breed difference in the expression of this protein. Conversely, in a comparison of control versus copper-challenged NR gels there was a 2-fold decrease in expression of the protein at high copper challenge. Methionine adenosyl transferase catalyses the formation of S-adenosylmethionine as part of the activated methyl cycle. Supplementation of ethanol fed rats with S-adenosylmethionine resulted in a 40–50% increase in the mitochondrial glutathione pool accompanied by mitochondrial protection [20]. A decrease in the expression of methionine adenosyl transferase in NR sheep liver as a result of copper challenge may result in a decrease in the availability of glutathione to the mitochondria and thus contribute to the oxidative stress-induced mitochondrial damage observed in NR livers [11].

**Figure 10:**
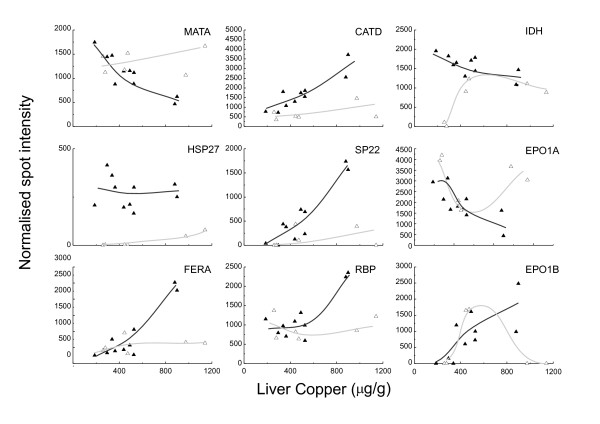
**Copper responsive protein expression: the relationship between normalised spot quantity and liver copper**. All 2-D gels in the study (3 replicates per animal) were assembled into one PDQuest matchset. Normalised protein spot quantities (in units of optical density ± SEM) for individual animals, both control and copper supplemented, are plotted against liver copper (μg/g, mean value of dorsal and ventral lobes). Symbols: Black and white triangles represent NR and Cambridge values respectively. Trend lines have been added manually to aid interpretation.

**Heat shock protein Hsp 27 **is a molecular chaperone able to modulate the ability of cells to respond to oxidative stress by maintenance of glutathione levels and by decreasing intracellular iron concentrations [21]. The expression of Hsp27 plays a role in TNFα-triggered signal transduction. The protein is also the effector protein of p38 MAP kinase in the p38 MAP kinase signalling pathway that mediates platelet derived growth factor-BB-stimulated migration of hepatic myofibroblasts [22]. Expression of Hsp27 was not copper responsive in NR sheep and was consistently higher than in Cambridge sheep (Figure 10). Hsp 27 was only detected in Cambridge sheep at high liver copper levels, indicative of copper responsive expression. Fibrosis is a pathological feature of copper toxicosis in NR [5,9] but not in domesticated breeds [8] therefore upregulation of Hsp27 in a pathway that mediates the migration of hepatic myofibroblasts which contribute to the development of fibrosis is an unexpected finding.

Several copper toxicoses in humans and animals are mutations of a specific carrier protein [23]. This occurs in Wilson disease with *WND *(*ATP7B*) expressed in the liver, the related *MNK *(*ATP7A*) in Menkes disease, expressed in the intestine and the *MURR1 *(*COMMD1*) deletion expressed in the liver of Bedlington terriers affected with copper toxicosis [24].

In humans, endemic Tyrolean infantile cirrhosis is genetically distinct from Wilson disease [25] and Bedlington terrier copper toxicosis [26]. Moreover, NR sheep do not to share the *MURR1 *deletion of the Bedlington terrier (Wijmenga, C. – unpublished data).

In humans it is increasingly recognized that differential expression of copper transporter proteins is time-dependent during the neonatal period. Copper absorption of humans at birth is high and reflects copper intake but declines soon after, in concert with the modulated expression of transporters Ctr1 and *ATP7A *[27]. Also liver copper concentrations are considerably higher in the foetus and neonate compared with adults and plasma caeruloplasmin is subnormal [28]. In the post natal period (3–6 m) there is an induction of biliary copper excretion and caeruloplasmin synthesis, presumably due to activation of the *ATP7B *(*WND*) gene. By comparison, in sheep foetal copper levels are lower than in adult sheep [29] and whilst there is no evidence of defective expression of *WND *(*ATP7B*), biliary copper excretion, but not caeruloplasmin synthesis, is impaired [30]. Recent work has identified different isoforms of WND protein in sheep which may explain the anomaly and by extension the sensitivity of sheep as a species to copper poisoning [31].

North Ronaldsay sheep accumulate copper in their livers to ten times the extent of Cambridge sheep [11]. This suggests that infantile copper importer mechanisms may be still operable having been selected by evolutionary pressure for a uniquely copper deficient environment. A similar maturational dysregulation of homeostatic mechanisms may also operate in the childhood condition although this must remain speculative.

Furthermore NR sheep are uniquely susceptible to copper resulting in a lower threshold of sensitivity and hence increased susceptibility to oxidative stress in which the mitochondria play a key role. Mitochondrial changes in the NR sheep have been contrasted with those in Wilson disease, Bedlington Terrier copper toxicosis and LEC rats in a companion paper [11]. However, some superficial similarities exist between the pathology of the mitochondria in NR hepatocytes and those of the toxic milk mouse, a murine model of Wilson disease [32,33] although the primary genetic defect in NR sheep has not been elucidated.

Interestingly, the yeast mitochondrion contains a dynamic pool of copper localised in the matrix as part of a low molecular weight anionic complex [34]. Expansion of this copper pool by the addition of copper salts to the growth medium did not result in any respiratory defects [34]. In contrast to the copper-challenged Cambridge sheep which showed minimal evidence of mitochondrial damage, ballooning and rupture of this organelle occurred in the NR sheep with an equivalent liver copper overload, which may suggest a failure to regulate this expandable copper pool resulting in the generation of reactive oxygen species and the pathological changes noted [11].

## Conclusion

A comparison of differential protein expression in response to copper challenge between the two breeds of sheep, complemented by ultrastructural studies, has affirmed the exquisite sensitivity of NR sheep to Cu-induced oxidative stress and suggest that this may arise from within mitochondria. Secondary consequences derived from the oxidative stress contribute to the overall pathology. The NR sheep is a unique model of altered or arrested copper homeostasis that will be of considerable value in the ontology of copper metabolism and its pathology in human infants and animals.

## Methods

### Experimental animals

Fourteen NR ewes aged 10 months were used in this study, of which six received a diet of hay (Cu 5 mg/kg dry weight) and 250 g/head/day of beet (Cu 5.4 mg/kg dry weight) over a six month period. The remaining animals were given the same diet except that the beet contained 15 mg/kg copper, a supplement of CuSO_4 _having been added. The copper-supplemented animals were killed in pairs at interval of 1, 2, 4 and 6 months after the commencement of the experiment, to achieve liver copper concentrations similar to those produced in Cambridge sheep [10]. The non-supplemented animals were killed in pairs at 1, 2 and 6 months.

Nine Cambridge ewes were housed and fed for a period as described above except that six animals received copper-supplemented pellets containing (Cu 155 mg/kg) and three animals received the same copper un-supplemented diet containing less than 50 mg/kg copper. With the basal diet of hay each animal received 500 g/day pelleted feed.

Ethical statement: The animal experiments were carried out under licence granted by the ANIMAL (SCIENTIFIC PROCEDURES ACT) 1986.

### Metal analysis

Sample preparation and analysis of liver tissue by inductively-coupled plasma mass spectrometry (ICP-MS) has been described previously [10].

### Electron microscopy

Full details of the method have been described previously [5,11].

### 2-DE of soluble liver proteins

Preparation of liver supernatants and their resolution and visualisation on 2-D gels has been described previously [10].

### Image analysis of 2-DE gels

Gels were scanned using an Epson 1680 pro flat bed scanner (266 dpi, 24-bit colour) and images were saved in an uncompressed TIFF file format. The gels were compared using the PD Quest 7.1 gel analysis software (Bio-Rad). Gels were normalised by reference to total intensity on the gel and replicate groups created from replicate gels (n = 3). Statistical analysis sets were created between two copper-challenged Cambridge and North Ronaldsay animals and statistically significant changes in proteins were identified by Student's t-test with a confidence level of 95%. Proteins were excised from the gels by the ProteomeWorks spot cutter (Bio-Rad). Excised gel plugs were robotically de-stained, digested with trypsin and spotted onto a MALDI target plate by a MassPREP Workstation (Waters, Manchester, UK).

### Characterisation of protein spots by MALDI-MS

Peptide mass fingerprinting of protein digestion products was performed on a M@LDI R mass spectrometer (Waters, Manchester, UK). Protein databases were searched using the Mascot search engine [35]. The monoisotopic masses of the tryptic peptides were compared to the Swiss-Prot and NCBI mammalian databases [36,37] using a mass tolerance of 150–200 ppm, allowing for up to one missed tryptic cleavage, and with carbamidomethylation of cysteine residues as a fixed modification and oxidation of methionine as a variable modification.

### Electrospray Ionisation Mass Spectrometry

Electrospray ionisation mass spectrometry (ESI-MS) and tandem mass spectrometry (ESI-MSMS) were performed on a Waters Q-ToF Micro instrument, fitted with a nanospray source. The electrospray was created from a silver-coated glass capillary with a 10 μm orifice (Presearch, Hitchin, UK), held at a potential of +2000 V relative to the sample cone. Prior to ESI-MSMS peptides were separated by reversed phase HPLC on a Dionex Ultimate system. The system was fitted with a PepMap C18 column (LC Packings, Camberley, Surrey, UK), 15 cm × 75 μm, bead size 3 μm and pore size 100Å. Prior to separation, the sample digest (20–25 μL) was taken up into the injection loop of the Dionex Famos autosampler and desalted in-line using a Dionex Switchos apparatus, fitted with a 5 mm × 300 μm, C18 precolumn. The precolumn was initially equilibrated in 0.2%(v/v) formic acid (solvent A) at 30 μL/min. Peptides were then loaded and washed for 3 min at the same flow rate, after which, the trap and downstream PepMap column were developed with 90% acetonitrile/0.2% formic acid (solvent B) introduced as a linear gradient of 0–50% in 30 min at 0.2 μL/min. The column eluant was monitored for uv absorbance at 214 nm in an in-line flow cell prior to being introduced into the mass spectrometer. The initial quadropole analyser was set to allow the transmission of selected precursors into the gas cell, where they were fragmented by collision with argon. The masses of the resulting fragment ions were then measured by the ToF analyser. Selection of precursors and fragmentation energy were controlled automatically using the data-dependent acquisition facility within the MassLynx software. Precursor spectra were acquired between m/z 400 and 1500 at a scan/interscan speed of 2.4/0.1 s. Product ion spectra were acquired between m/z 100 and 2000 at a scan/interscan speed of 1.0/0.1 s. Raw product ion spectra were deconvoluted using the MaxEnt 3 alogorithm within the MassLynx software. The charge state of the parent peptide was determined from the isotope envelope in the precursor spectrum. Interpretation of product ion spectra and the determination of peptide sequences were facilitated by the PepSeq module within MassLynx. Peptide sequences were searched using Blast (Basic Local Alignment Search Tool) [37].

### Immunohistochemical analysis of tissue microarrays

Tissue microarrays (24 × 2 mm cores) were prepared from formalin-fixed paraffin-embedded liver (dorsal and ventral lobes) using a Tissue Micro Array Builder (Abcam, Cambridge, UK). Ultrathin sections from the donor block were mounted on charged slides permitting the simultaneous screening of tissue from a number of animals with antibodies. The TMAs were de-waxed in xylene and re-hydrated by immersion in a series of alcohol, followed by water baths. Endogenous peroxide activity was blocked using the peroxidase solution provided with the Envision dual labelling kit (DakoCytomation, Cambridge, UK). The TMA slides were washed in Tris-buffered saline (TBS) pH 7.6 0.05% (v/v) Tween 20 before incubation with 1% (w/v) BSA in TBS pH 7.6 for 1 hr at room temperature. After removal of the blocking solution the TMAs were incubated with a 1:250 dilution (~200 μL/slide) of anti-metallothionein antibody (E9 monoclonal, DakoCytomation) in TBS pH 7.6 overnight at 4°C. TMA slides were then developed further using the Envision^®+ ^Dual link system. A control slide was included in the experiment and was incubated in blocking solution overnight. The slides were given 3 × 5 min washes in TBS/0.05%(v/v)Tween then incubated with a peroxidase-labelled polymer conjugated to goat anti-mouse and goat anti-rabbit immunoglobulins included with the kit, for 30 min at room temperature. After a further 3 washes, as above, the TMAs were developed using the diaminobenzidine (DAB) buffered substrate provided with the kit. Colour development was stopped by immersing the slides in TBS-Tween containing 0.05%(w/v) azide, slides were counterstained with haematoxylin.

## Authors' contributions

DS carried out the comparative gel analysis and mass spectral analysis and identification of differentially expressed proteins in addition to drafting this manuscript. SH participated in the design of this study, carried out ultrastructural studies on the sheep liver and was involved in the writing and critical evaluation of this manuscript. RJB conceived the study, participated in its design and critically evaluated this manuscript. RJB is also responsible for updating and maintenance of in-house bioinformatics. AM set up the tissue microarray study and assisted in the interpretation of data. All authors read and approved the final manuscript.

## Supplementary Material

Additional file 1 **A Two-dimensional gel of soluble liver proteins isolated from Cambridge and North Ronaldsay sheep following high copper challenge**. Soluble liver proteins were isolated from the livers of NR and Cambridge sheep following high copper challenge. The proteins were separated in the first dimension across the pH range 5–8 followed by resolution on linear12.5% (w/v) polyacrylamide gels. Spot detection was carried out using Phoretix 2D Evolution Software followed by warping of the images to align similar features and to allow differences between gels to be seen more easily. Viewing the images using the warp facility allows identification of regions or spots that are significantly different between the two images. The single green spots indicate proteins that are present only in the NR and magenta spots are those proteins only present in Cambridge gels at a particular location. Click here for file

Additional file 2 **Non-copper-responsive proteins: breed-specific differences in protein expression identified between Cambridge and North Ronaldsay sheep**. 2-D gels of soluble proteins isolated from NR and Cambridge sheep liver at high copper challenge were run in triplicate and placed in analysis sets using PDQuest 2-D gel analysis software. Statistical analysis sets were created from two copper-challenged Cambridge and North Ronaldsay animals. 2-D gels from each animal were run in triplicate and statistically significant changes in protein expression were identified by Student's t-test at p ≤ 0.05. Proteins in the list were flagged as being statistically different between Cambridge and NR gels. With reference in each case to proteins spots from gels of control, non-supplemented animals run in parallel, a list of proteins whose expression differs between NR and Cambridge sheep as a result of breed difference has been compiled. Click here for file
