# Supplementary figures and images for: A proteomics study of the response of North Ronaldsay sheep to copper challenge
Source: BMC Vet Res. 2006 Dec 27;2:36. doi: 10.1186/1746-6148-2-36 (PMC1766353; doi:10.1186/1746-6148-2-36)

## Slide 1
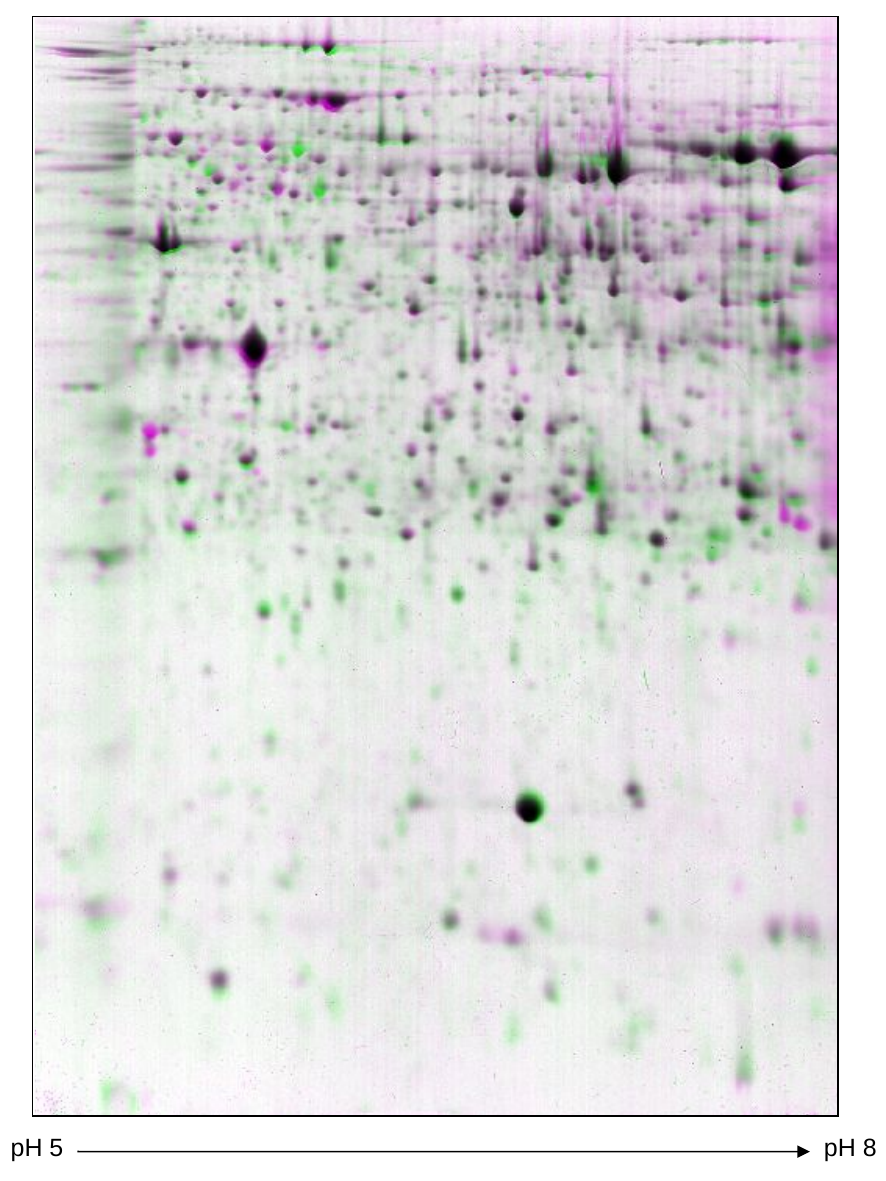

pH 5
pH 8

Supplement: Additional file 1 — A Two-dimensional gel of soluble liver proteins isolated from Cambridge and North Ronaldsay sheep following high copper challenge. Soluble liver proteins were isolated from the livers of NR and Cambridge sheep following high copper challenge. The proteins were separated in the first dimension across the pH range 5–8 followed by resolution on linear12.5% (w/v) polyacrylamide gels. Spot detection was carried out using Phoretix 2D Evolution Software followed by warping of the images to align similar features and to allow differences between gels to be seen more easily. Viewing the images using the warp facility allows identification of regions or spots that are significantly different between the two images. The single green spots indicate proteins that are present only in the NR and magenta spots are those proteins only present in Cambridge gels at a particular location. [file 1746-6148-2-36-S1.ppt]
